# Differential microbiome features in lake–river systems of Taihu basin in response to water flow disturbance

**DOI:** 10.3389/fmicb.2024.1479158

**Published:** 2024-09-30

**Authors:** Peng Xiao, Yao Wu, Jun Zuo, Hans-Peter Grossart, Rui Sun, Guoyou Li, Haoran Jiang, Yao Cheng, Zeshuang Wang, Ruozhen Geng, He Zhang, Zengling Ma, Ailing Yan, Renhui Li

**Affiliations:** ^1^National and Local Joint Engineering Research Center of Ecological Treatment Technology for Urban Water Pollution, Zhejiang Provincial Key Lab for Water Environment and Marine Biological Resources Protection, Wenzhou University, Wenzhou, China; ^2^CCCC Shanghai Waterway Engineering Design and Consulting Co., Ltd, Shanghai, China; ^3^Department of Plankton and Microbial Ecology, Leibniz-Institute of Freshwater Ecology and Inland Fisheries (IGB), Stechlin, Germany; ^4^Institute of Biochemistry and Biology, University of Potsdam, Potsdam, Germany; ^5^College of Life Sciences and Technology, Harbin Normal University, Harbin, China; ^6^Research Center for Monitoring and Environmental Sciences, Taihu Basin & East China Sea Ecological Environment Supervision and Administration Authority, Ministry of Ecology and Environment of the People’ s Republic of China, Shanghai, China; ^7^Shanghai Engineering Research Center of Water Environment Simulation and Ecological Restoration, Shanghai Academy of Environment Sciences, Shanghai, China

**Keywords:** water flow disturbance, river–lake system, microbial community, microeukaryote, community assembly, Lake Taihu

## Abstract

**Introduction:**

In riverine ecosystems, dynamic interplay between hydrological conditions, such as flow rate, water level, and rainfall, significantly shape the structure and function of bacterial and microeukaryotic communities, with consequences for biogeochemical cycles and ecological stability. Lake Taihu, one of China’s largest freshwater lakes, frequently experiences cyanobacterial blooms primarily driven by nutrient over-enrichment and hydrological changes, posing severe threats to water quality, aquatic life, and surrounding human populations. This study explored how varying water flow disturbances influence microbial diversity and community assembly within the interconnected river–lake systems of the East and South of Lake Taihu (ET&ST). The Taipu River in the ET region accounts for nearly one-third of Lake Taihu’s outflow, while the ST region includes the Changdougang and Xiaomeigang rivers, which act as inflow rivers. These two rivers not only channel water into Lake Taihu but can also cause the backflow of lake water into the rivers, creating distinct river–lake systems subjected to different intensities of water flow disturbances.

**Methods:**

Utilizing high-throughput sequencing, we selected 22 sampling sites in the ET and ST interconnected river-lake systems and conducted seasonally assessments of bacterial and microeukaryotic community dynamics. We then compared differences in microbial diversity, community assembly, and co-occurrence networks between the two regions under varying hydrological regimes.

**Results and discussion:**

This study demonstrated that water flow intensity and temperature disturbances significantly influenced diversity, community structure, community assembly, ecological niches, and coexistence networks of bacterial and eukaryotic microbes. In the ET region, where water flow disturbances were stronger, microbial richness significantly increased, and phylogenetic relationships were closer, yet variations in community structure were greater than in the ST region, which experienced milder water flow disturbances. Additionally, migration and dispersal rates of microbes in the ET region, along with the impact of dispersal limitations, were significantly higher than in the ST region. High flow disturbances notably reduced microbial niche width and overlap, decreasing the complexity and stability of microbial coexistence networks. Moreover, path analysis indicated that microeukaryotic communities exhibited a stronger response to water flow disturbances than bacterial communities. Our findings underscore the critical need to consider the effects of hydrological disturbance on microbial diversity, community assembly, and coexistence networks when developing strategies to manage and protect river–lake ecosystems, particularly in efforts to control cyanobacterial blooms in Lake Taihu.

## 1 Introduction

Running water systems, such as rivers (lotic ecosystems), and still water systems, such as lakes (lentic ecosystems), are key components of inland water ecosystems, playing essential roles in various biogeochemical processes on Earth (Battin et al., 2023; Maavara et al., 2020). Hydrological conditions including flow rate, water level, hydraulic retention time, and rainfall critically influence nutrient cycling and eutrophication risks, thereby affecting the structure and resilience of bacterial and microeukaryotic communities and potentially leading to loss of biodiversity (Chen et al., 2023; Shabarova et al., 2021; Zhang L. et al., 2021a). These changes add uncertainty to predictions and management of ecological environments, underscoring the need to understand microbial responses to varying hydrological conditions (Palmer and Ruhi, 2019).

Bacterial communities in riverine waters are susceptible to hydrological connectivity across different watershed scales, which influences nutrient availability, pollution, and environmental parameters such as temperature and light (Fang et al., 2023; Niu et al., 2024; Zhang et al., 2022). Fluctuations in flow velocity and water levels shift nutrient availability and weaken algal bloom development (Chen et al., 2023; Hu et al., 2024; Yang et al., 2017, 2022), while rainfall and water flow can introduce pathogens and other bacteria from terrestrial runoff and sewage, impacting the structure and interactions within microbial communities, especially in riverine systems (Kim et al., 2017; Wilkes et al., 2009; Yu et al., 2023). Changes in flow rate, reflecting variations in water source, residence time, and streambed contact, can lead to substantial shifts in microbial diversity and community structure (Bambakidis et al., 2024; Clark et al., 2022; Niño-García et al., 2016; Wang et al., 2016).

Although significant, regional variations in microbial responses to hydrological changes are still rarely understood, highlighting the urgency for more integrated studies on the synergistic effects of hydrological and environmental factors across various spatiotemporal scales. Deterministic processes, such as heterogeneous and homogeneous selection, emphasize the role of environmental factors and microbial interactions within niche theory frameworks. In contrast, stochastic processes, including dispersal limitation, homogenizing dispersal, and drift, suggest that community assembly is influenced by random factors according to neutral theory (Ning et al., 2020; Stegen et al., 2013; Zhou and Ning, 2017). Analyzing microbial community assembly under fluctuating flow conditions elucidates ecological processes that govern biodiversity and ecosystem functioning, improving strategies for ecosystem management and conservation.

Microeukaryotic communities play diverse and critical roles within river ecosystems, including nutrient cycling, organic matter decomposition, and serving as both primary producers and consumers within food webs. They are influenced by environmental factors across different spatial and temporal scales, reflecting their short reproductive cycles, broad distribution, and high sensitivity to pollutants (Tamminen et al., 2022; Xu et al., 2020; Yang et al., 2023). Apart from microeukaryotes, bacterioplankton is a driver of biogeochemical cycles, such as nutrient cycling and degradation of pollutants in rivers (Li Y. et al., 2023a; Liu et al., 2018; Zhang B. et al., 2021b). Despite extensive research on the gradients and diversity of these communities in running waters, little attention has been given to their dynamics due to fluctuating water flow disturbances. This is surprising as they hold significant roles in aquatic productivity, food webs, and various biogeochemical processes. Differences among microbial domains, suggested by initial evidence from aquatic systems, can be substantial (Blais et al., 2024; Siriarchawatana et al., 2024; Zhang L. et al., 2021a). Furthermore, studies revealed that anthropogenic activities and rainfall introduce nutrients that significantly alter the riverine microeukaryotic rather than bacterial communities (Liu et al., 2021; She et al., 2023). Consequently, there is a critical need to improve our comparative understanding of the underlying mechanisms leading to such differences between microeukaryotes and bacteria in riverine ecosystems.

Located in the middle and lower reaches of the Yangtze River, the Yangtze River Delta functions as both an economic indicator for the Yangtze River Economic Belt (YREB) and an ecological barrier to the sea (Chen et al., 2017). Lake Taihu, the largest freshwater lake in YREB, has experienced significant ecological challenges in recent years, particularly the frequent occurrence of cyanobacterial blooms. These blooms, which are primarily driven by nutrient over-enrichment and hydrological alterations, pose severe threats to the lake’s water quality, aquatic ecosystems, and the health of surrounding human populations (Qin et al., 2019). As a central hub of complex river–lake interactions characterized by substantial organismic and material exchange, Lake Taihu’s ecological balance is highly sensitive to these environmental stressors, making the study of such dynamics critical for the region’s environmental management and sustainability (Lai et al., 2013). The lake receives inflows from over 200 rivers, including 30 major ones, contributing to a vast water network that spans 120,000 km^2^ across the Lake Taihu basin, averaging 3.2 km of river length per square kilometer of land (Li et al., 2021). The Taipu River, a key flood channel that connects Lake Taihu to the downstream Huangpu River, plays a pivotal role within the YREB’s green and integrated ecological development zone (Shao et al., 2023).

Internationally, inter-regional water diversion projects are commonly employed for irrigation, flood control, water supply, and power generation (Duan et al., 2022). Such projects often involve the introduction of large volumes of clean water to combat eutrophication in lakes and reservoirs, a practice adopted by numerous countries (Eamen et al., 2021; Larsen et al., 2016; Long et al., 2024). Recently, the Yangtze River to Lake Taihu water diversion project has shortened water residence times in the lake, significantly affecting its hydrology by increasing its water level fluctuations and the flood discharge capacity of the Taipu River in the lake’s eastern region (Hu et al., 2021; Li et al., 2013; Zhai et al., 2010; Zhang et al., 2023a). In the southern part of the lake, backflow into the Changdougang and Xiaomeigang rivers in Huzhou City is frequent (Xu et al., 2014), highlighting the dynamic hydrological and hydrodynamic shifts that can substantially influence environmental heterogeneity and microbial dispersal capabilities. Despite, responses of bacterial and microeukaryotic communities to varied hydrological disturbance and their underlying mechanisms remain little understood.

To address this gap, we conducted a high-throughput sequencing investigation of bacterial and microeukaryotic communities within the interconnected East (ET) and South (ST) river–lake systems of Lake Taihu. Our objectives were to (1) delineate the composition and distribution of these microbial communities under different flow conditions in the ET and ST regions; (2) assess and compare their diversity, community assembly, and interspecies coexistence across the water flow-induced environmental gradients; and (3) explore the potential impacts of varying intensities of flow disturbances on these microbial communities. Thereby, we seek to enhance our understanding of flow-induced changes in microbial dynamics within these critical freshwater ecosystems.

## 2 Materials and methods

### 2.1 Sample collection

Lake Taihu, fed by the Yangtze River, is China’s third largest freshwater lake. It is located in one of the most developed areas of China, the Yangtze River Delta. The Taipu River, located in the southeast of Lake Taihu, is one of the lake’s largest outflow rivers, accounting for nearly 2/3 of its total outflow. It is also a crucial source of drinking water for downstream provinces and cities, including Zhejiang, Jiangsu, and Shanghai City, supplying drinking water to nearly 20 million people. It thus plays an important role in regulating and supplying water in eastern China (Yan et al., 2024). Both the Changdougang River and Xiaomeigang River are located in the southwest of Lake Taihu. These inflow rivers not only discharge water from Lake Taihu but can also hold the potential to channel water back into the lake when their water levels are relatively high. In contrast, the Taipu River exclusively draws water from Lake Taihu. The Taipu River in the ET region accounts for nearly one-third of Lake Taihu’s outflow. In contrast, the rivers Changdougang and Xiaomeigang in the ST region contribute to only a small portion of the outflow and transport water into Lake Taihu during heavy rainfall. While rainfall between the two regions showed no significant difference, discharge did.

In winter (February) of 2022 and spring (May), summer (August) and autumn (November) of 2023, surface water samples (upper 0.5 m) were collected from 22 sites, totaling 88 samples. These included four sites in the south of Lake Taihu, seven inflow river sites into Lake Taihu (four at Changdougang River and three at Xiaomeigang River), three sites in the east of Lake Taihu, and eight outflow river sites from Lake Taihu (all at Taipu River). Water temperature (WT), pH, dissolved oxygen (DO), and electrical conductivity (EC) were also measured *in situ* using a multi-parameter probe system (Hach Company, Loveland, CO, USA). The water transparency (SD) was measured using a Secchi disk. One liter of surface water was sampled at each site and sealed in glass bottles for further analysis of total nitrogen (TN), ammonium nitrogen (NH_4_N), nitrate nitrogen (NO_3_N), total phosphorus (TP), phosphate phosphorus (PO_4_P), permanganate index (COD_Mn_), and chlorophyll-*a* (Chl*a*) according to the standard methods described in our previous study (Mo et al., 2021). Approximately 500 mL of each water sample were filtered through 0.22 μm filters (GTTP, Millipore, Billerica, MA, USA) for microbial community analyses. Filters were then stored at −80°C for DNA extraction. One liter of surface water sample was preserved with Lugol’s iodine solution. After sitting for at least 24 h, phytoplankton (Phy_biomass) and cyanobacteria (Cya_biomass) identification and biomass quantification were performed using an optical microscope (DM2000, Leica Microsystems, Deerfield, IL, USA) according to our previous study (Li et al., 2020). The rainfall data (Rain_fall) for both South and East Lake Taihu regions, the net inflow water volume of Lake Taihu (Inflow), and the outflow discharge data of rivers (Outflow) in the study area were downloaded from the website of the Taihu Basin Authority of the Ministry of Water Resources^1^. The spatial distribution maps were improved using ArcMap 10.5 (ESRI, Redlands, USA).

### 2.2 DNA extraction, PCR, and Illumina sequencing

DNA extraction from the filters were performed using the FastDNA Spin kit (MP Biomedicals, Santa Ana, CA, USA) according to the manufacturer’s instructions. For the prokaryotic microbial community, the V3-V4 hypervariable regions of the 16S rRNA gene were amplified using the primers 341F and 806R following our previous procedure (Herlemann et al., 2011; Zuo et al., 2023). For microeukaryotic microbial communities, the V9 hypervariable region of the 18S rRNA gene was amplified using primers 1380F and 1510R (Amaral-Zettler et al., 2009). The PCR reaction mixture included 10 μL of high-fidelity Taq PCR Mix (Takara, Dalian, China), 0.2 μM of each primer, approximately 10 ng of sample DNA, and sterile ddH_2_O to yield a final volume of 20 μL. The PCR protocol consisted of an initial denaturation at 95°C for 2 min, followed by 30 cycles of 95°C for 30 s, 55°C for 30 s, and 72°C for 30 s, with a final extension at 72°C for 10 min. The PCR products from three replicates per sample were combined and subjected to agarose gel electrophoresis. The PCR products were then sequenced on an Illumina NovaSeq platform using PE250 (prokaryotic) and PE150 (microeukaryotic) sequencing.

### 2.3 Bioinformatics

The sequenced raw reads were processed using the dada2 package in R (v4.10^2^), including primer and adapter removal, quality filtering, and chimera removal (Callahan et al., 2016). Bacterial and microeukaryotic ASVs (amplicon sequence variants) were annotated using the Silva v138.1 (Quast et al., 2012) and PR2 v5.0.0 (Guillou et al., 2012) databases, respectively, with the sintax algorithm in the Vsearch v2.28.1 software (Rognes et al., 2016). After removing non-target sequences, the prokaryotic and microeukaryotic microbial communities were normalized to 18,984 and 134,852 reads across all samples, respectively, using the vegan package in R v4.10.

### 2.4 Statistical analysis

Phylogenetic trees of bacterial and microeukaryotic ASVs were constructed using the align-to-tree-mafft-fasttree module of QIIME2. The richness and Bray-Curtis diversity indices were calculated using the core-metrics-phylogenetic module of the QIIME2 platform (Bolyen et al., 2019). Non-metric multidimensional scaling (NMDS), analysis of similarity statistics (ANOSIM), and the non-parametric Kruskal–Wallis test were performed to assess group significance using the “vegan” package in R v4.10.

Community distance-decay relationships were linearly fitted based on geographical and dendritic distances. The Local Contribution to Beta Diversity (LCBD) was computed using the “adespatial” package in R v4.10 to quantify the contributions of the 22 sample sites to the overall diversity of bacterial and microeukaryotic communities in the interconnected river–lake system of South and East Lake Taihu (Legendre and De Cáceres, 2013). The correlation between microbial community (Bray-Curtis distance) and environmental factors was assessed using the Mantel test with the “linkET” package^3^. Hierarchical partitioning (HP) calculated the proportion of environmental factors explaining microbial community variation in ET and ST habitats using the “rdacca.hp” package (Lai et al., 2022).

The nearest taxon index (NTI), calculated by using the “picante” package, represents the standardized effect size of the mean nearest taxon distance (MNTD) in the community, indicating phylogenetic clustering (positive NTI) or overdispersion (negative NTI). An NTI of 0 signifies random phylogenetic relationships (Webb, 2000). The normalized stochasticity ratio (NST), *β*-nearest taxon index (βNTI), and the Bray-Curtis-based Raup-Crick metric (RC_bray_) were calculated using the NST package (Ning et al., 2019). The fit of the neutral community model was assessed using the “Hmisc” package (Chen et al., 2019). Habitat niche width and overlap of bacterial and microeukaryotic communities were calculated using the “spaa” package following our previous study (Zuo et al., 2023).

Co-occurrence networks were constructed based on robust Spearman correlations (|*r*| > 0.8, *p* < 0.01) and RMT analysis (Feng et al., 2022; Xue et al., 2018). ASVs, occurring in <50% of all samples, were removed to reduce rare ASVs. Networks were constructed using the “igraph” package (Csardi and Nepusz, 2005) and visualized with Gephi v0.9.2 (Bastian et al., 2009). Module hubs (Zi-score > 2.5, Pi-score < 0.62), connectors (Zi-score < 2.5, Pi-score > 0.62), and peripherals (Zi-score < 2.5, Pi-score < 0.62) were identified (Strogatz, 2001). Additionally, 1,000 Erdös-Rényi random networks, matching real networks in nodes and edges, were generated using the “igraph” package. Topological characteristics, including modularity, clustering coefficient, and average path length, were compared between real and random networks with Gephi.

We utilized partial least squares path modeling (PLS-PM) to analyze the relationships among water temperature (WT), hydrology, physicochemical factors, microbial richness, microbial phylogenetic diversity, ecological niches, and composition of both bacterial and microeukaryotic communities in the ET and ST regions. The PLS-PM analysis was performed using the ‘plspm’ package (Tenenhaus et al., 2005). Random forest (RF) machine learning assessed the effects of environmental factors on alpha-and beta-diversity indices (Breiman, 2001). Checkerboard score (C-score) analysis was conducted to test clustering or overdispersion of microbial communities using the “EcoSimR” package, following our previous study (Mo et al., 2021).

## 3 Results

### 3.1 Physicochemical properties of the east and south regions of Lake Taihu (ET&ST)

**Figure 1:**
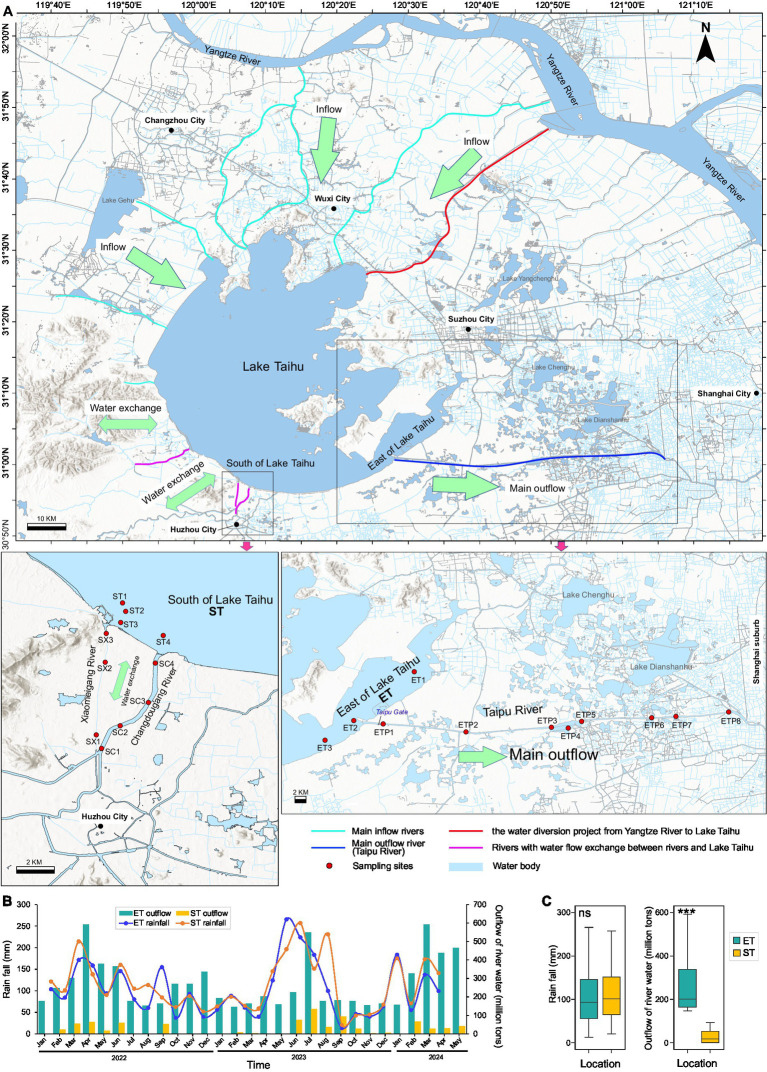
Locations and hydrological characteristics of the study area. **(A)** Locations of sampling sites. Bold arrows indicate major inflow and outflow regions of Lake Taihu. ST represents the South Taihu river–lake system, and ET represents the East Taihu rive-laker system. **(B)** Monthly cumulative rainfall and outflow water volume for the two regions from 2022 to 2024. **(C)** Monthly rainfall and outflow water volume in both locations during the sampling period. ***p < 0.001; Kruskal–Wallis test; ns, non-significant.

The river–lake interconnected ET and ST regions are located in the southeastern and southwestern parts of the lake, respectively (Figure 1A). The monthly average rainfall between the ET and ST regions showed no significant difference. However, there was a significant difference in the monthly average discharge into the lake, with the ET region’s discharge (165 ± 15 million tons per month) being nearly ten times that of the ST region (19 ± 17 million tons per month). Other environmental factors between the two regions did not show significant differences, except for NO_3_N, SD, and EC. The average values in the ET region were 0.69 (0.17–1.75) mg/L for NO_3_N, 50.23 (13–76) cm for SD, and 429.07 (279–572) μS/cm for EC, compared to the ST region’s averages of 0.45 (0.04–0.98) mg/L, 36.84 (13–60) cm, and 345.20 (216–498) μS/cm, respectively (Figures 1B,C).

### 3.2 Microbial diversity in ET vs. ST regions

**Figure 2:**
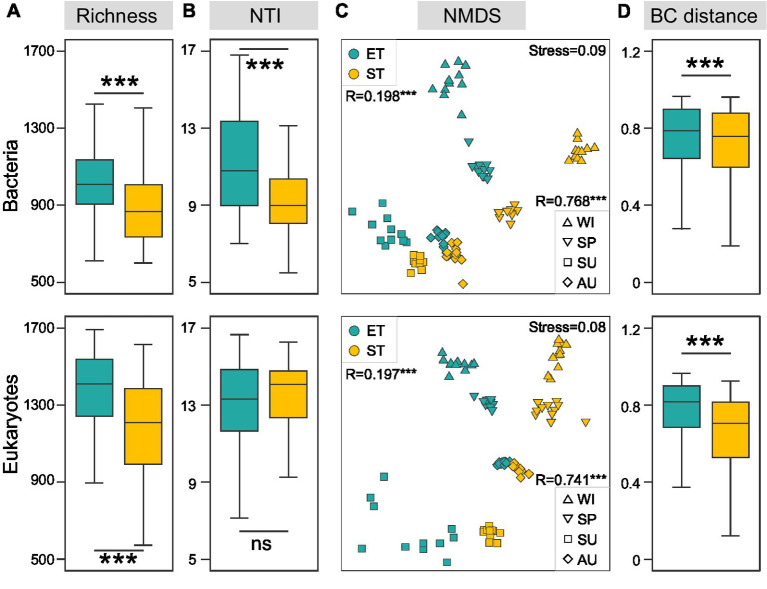
Regional variations in bacterial and micro-eukaryotic communities in the two river–lake systems. Richness **(A)** and nearest taxon index (NTI, **B**) of both regions. **(C)** Ordination analysis of communities using non-metric multidimensional scaling (NMDS) based on Bray-Curtis dissimilarities (***p < 0.001, non-parameter Kruskal–Wallis test; ns, non-significant). **(D)** *R*-values from ANOSIM analyses between different regions are displayed in the top left corner, while the *R*-values from ANOSIM analyses between different seasons are shown in the bottom right corner (***p < 0.001). Regional variation of Bray-Curtis distance (***p < 0.001, non-parameter Kruskal–Wallis test).

We analyzed a total of 1,670,592 bacterial and 11,866,976 microeukaryotic quality-filtered sequences across all samples, grouped into 13,464 and 17,552 ASVs, respectively. Rarefaction curves for both bacterial and microeukaryotic richness reached saturation (Supplementary Figure S1), suggesting comprehensive retrieval of ASVs. Bacterial and microeukaryotic community richness was significantly higher in the ET than in the ST region. Furthermore, NTI diversity for bacterial communities was significantly higher in ET than in ST, although NTI diversity for microeukaryotic communities showed no significant regional differences (Figures 2A,B). These results suggested that the ET region, which experienced higher water flow disturbances, harbored a greater diversity of bacterial and eukaryotic species that exhibited closer phylogenetic relationships, indicating a potential influence of species sorting when compared to the ST region. Using NMDS analysis, we observed significant seasonal variations in the spatiotemporal dynamics of both bacterial and microeukaryotic communities in both ET and ST regions (ANOSIM R values were 0.768 and 0.741, respectively; both *p-*values were 0.001). The ANOSIM analysis results suggested that seasonal variation was more pronounced in the ST region than in the ET region (Supplementary Figure S2). Additionally, significant differences were noted between the bacterial and microeukaryotic communities of the ET and ST regions (ANOSIM R values were 0.198 and 0.197, respectively; both *p* values were 0.001) (Figure 2C), with higher dissimilarities observed in the ST region (Figure 2D).

Bacterial and microeukaryotic ASVs were classified into 69 and 50 phyla/supergroups, respectively. Proteobacteria was the most prevalent bacterial phylum in both the ET and ST regions, comprising 40.03 ± 13.12% in ET and 36.53 ± 10.83% in ST. In ET, the next most abundant bacterial phyla were Actinobacteria (24.81 ± 10.96%), Bacteroidota (10.83 ± 12.47%), and Cyanobacteria (9.54 ± 10.45%). Similarly, in ST, Actinobacteria (30.43 ± 9.35%) were followed by Bacteroidota (10.89 ± 6.86%) and Cyanobacteria (12.59 ± 8.53%). For microeukaryotes, the ET region had high abundances of Gyrista (33.27 ± 14.12%), Metazoa (18.75 ± 15.41%), Chlorophyta (12.55 ± 6.77%), and Fungi (11.01 ± 9.82%), whereas in ST, Gyrista (30.15 ± 11.50%), Metazoa (21.84 ± 13.41%), Cryptophyta (9.57 ± 4.15%), and Fungi (7.93 ± 5.43%) were prevalent (Supplementary Figure S3). Additionally, the cyanobacterial biomass, predominantly composed of *Microcystis* and *Dolichospermum* species, did not differ significantly between the ET and ST regions; however, it was significantly higher in Lake Taihu than in the adjacent riverine waters (Supplementary Table S1).

### 3.3 Microbial community assembly processes and potential environmental drivers

**Figure 3:**
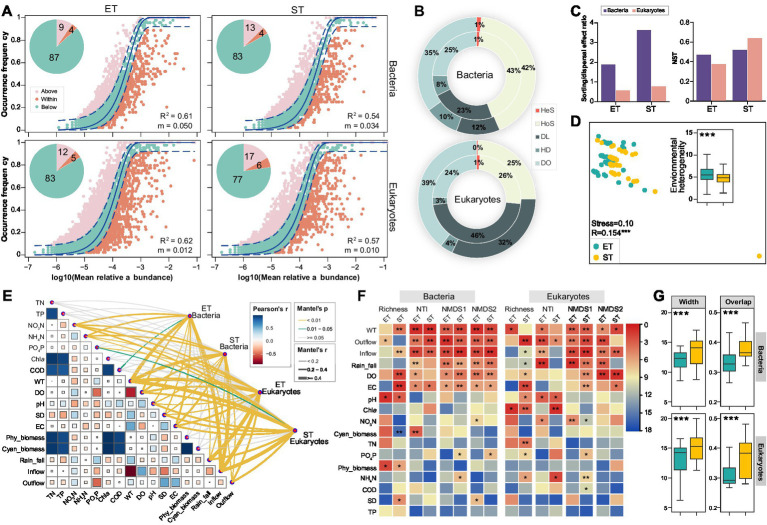
Bacterial and microeukaryotic community assembly and environmental divers in both interconnected river–lake systems. **(A)** Fit of neutral community model (NCM) for analysis of bacterial and microeukaryotic community assembly processes. Solid line represents the best-fitting neutral model; dashed lines represent the 95% CIs around the best-fitting neutral model. ASVs that occur more or less frequent than predicted by NCM are shown in different colors. *R^2^* indicates the fit to this model, and *m* indicates the immigration rate. Proportions (%) of the three groups of ASVs are displayed by pie charts. **(B)** Relative importance of different ecological processes based on iCAMP analysis for bacterial and microeukaryotic communities in the ET (inner rings) and ST (outer rings) regions: homogeneous selection (HoS), heterogeneous selection (HeS), dispersal limitation (DL), homogenizing dispersal (HD), drift and others (DO). **(C)** Sorting/dispersal effect ratio and normalized stochasticity ratio (NST) obtained based on null model analysis. **(D)** Non-metric multidimensional scaling (NMDS) ordinations revealing the environmental heterogeneity between ET and ST regions. ANOSIM test *R* value is shown, ***p = 0.001. Insert: overview of pairwise Euclidean distance of environmental factors in the ET and ST regions. All factors were normalized. **(E)** Environmental drivers of bacterial and microeukaryotic communities, evaluated by Mantel tests in the ET and ST regions. **(F)** Contribution of physicochemical properties to alpha (richness and NTI) and beta (NMDS axes based on Bray-Curtis dissimilarities) diversities of microbial communities based on random forest (RF) algorithm. Colors represent the rank of important variables based on the mean squared error (MSE) values (*p < 0.05, **p < 0.01). **(G)** Habitat niche widths (Width) and overlap (Overlap) of bacterial and microeukaryotic communities in the ET and ST regions (***p < 0.001; non-parameter Kruskal–Wallis test).

We applied the neutral community model (NCM) to assess the impact of stochastic processes on bacterial and microeukaryotic community assembly in ET and ST regions. The quality fit (*R*^2^) for all communities exceeded 0.5, demonstrating a significant influence of stochastic processes in both regions. Specifically, *R*^2^ and migration rates (m) were higher in the ET than in the ST region, indicating greater stochasticity and dispersal capability of microbial taxa in the ET region. In contrast, taxa exceeding NCM predictions occurred less frequently in bacterial and microeukaryotic communities in the ET than in the ST region (Figure 3A). The null model revealed that homogenizing selection primarily shaped bacterial communities, followed by dispersal limitation in both regions, with the opposite pattern in microeukaryotic communities. Dispersal limitation had a more pronounced effect on both microbial communities in the ET than in the ST region, indicating that microbial communities in the ET region were more influenced by dispersal limitations. Furthermore, unexplained community assembly processes were more prevalent in the ST region (Figure 3B). Stochastic contributions to community assembly were also found to be higher in the ET region for both microbial communities, as confirmed by the sorting/dispersal effect ratio and normalized stochasticity ratio (NST) analysis (Figure 3C). Additionally, in the ET region, the explanatory power of environmental factors for both bacterial and microeukaryotic communities was lower than in the ST region based on hierarchical partitioning analysis (Supplementary Figure S4), implies that both bacterial and microeukaryotic communities in ET region were less influenced by the measured environmental variables than those in ST region.

We also assessed the heterogeneity of physicochemical factors in the ET and ST regions, revealing significant regional differences (ANOSIM R = 0.154, *p* = 0.001; Figure 3D) with a notably higher variation in the ET region. To identify the environmental drivers of bacterial and microeukaryotic distribution, we correlated both alpha and beta diversity indices with differences in the physicochemical properties using Mantel tests and random forest analyses. Water temperature (WT), inflow, outflow, and rainfall emerged as strong, positive predictors for the observed variations in microbial taxa of the two regions (Figures 3E,F and Supplementary Figure S4). In both ET and ST regions, positive correlations were observed between microbial community dissimilarity and WT, Outflow, Inflow, and Rain_fall, implying that alterations in these environmental factors also increased the heterogeneity of microbial communities. Among all measured factors, Outflow had the most significant effect, which was higher in the ET than in the ST region for both bacterial and microeukaryotic communities (Supplementary Figure S5). Further, both bacterial and microeukaryotic communities exhibited significantly narrower niche width and overlap in the ET region than in the ST region (Figure 3G). More importantly, the C-score showed that the value of standardized effect size (SES) increased more in the ST than in the ET region in both bacterial and microeukaryotic communities. This indicated that the community assembly was more strongly influenced by deterministic processes in the ST than in the ET region (Supplementary Table S2). In addition, the contributions of different local communities to microbial diversity in the ST and ET regions were evaluated by Local Contribution to Beta Diversity (LCBD) analysis. In the ET region, throughout three sampling seasons of the year (excluding spring), both bacterial and microeukaryotic communities at upstream of Lake Taihu sampling points contributed more to community beta diversity than downstream river points. Conversely, in the ST region, no such trend was observed for either bacterial or microeukaryotic communities (Supplementary Figure S6). These results suggested that in the ET region, where the outflow direction was constant, headwater May significantly influence microbial community diversity. In contrast, in the ST region, where the flow direction repeatedly changed, the impact of Lake Taihu end and the river end on microbial community diversity was weaker.

### 3.4 Co-occurrence networks and stability of bacterial and microeukaryotic communities in the ET vs. ST regions

**Figure 4:**
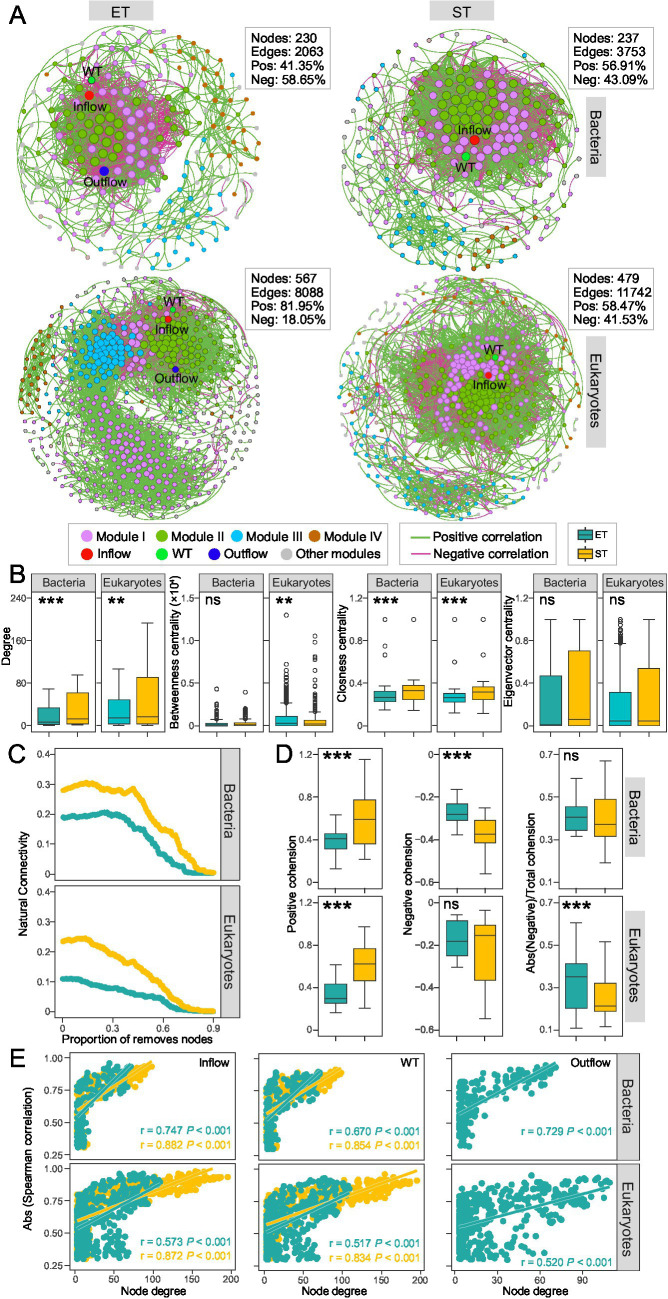
Bacterial and microeukaryotic co-occurrence network analysis. **(A)** Networks analysis revealing the significant Spearman’s correlations (|*r*| > 0.80, *p* < 0.01) between bacterial and microeukaryotic ASVs and environmental factors in ET and ST integrated networks. **(B)** Node-level topological characteristic parameters (***p < 0.001; **p < 0.01; ns, non-significant non-parameter Kruskal–Wallis test). **(C)** Network stability for bacteria and microeukaryotes in the ET and ST regions. **(D)** Positive and negative cohesion of microbes between the ET and ST regions. **(E)** Spearman’s correlations showing the significant relationship between the degree of connectedness of bacterial and microeukaryotic ASVs in the integrated network and their spearman’s correlation coefficients with Inflow (million tons per month), WT (°C) and Outflow (million tons per month) in the ET and ST regions.

We constructed and analyzed distinct microbial co-occurrence networks based on Spearman correlations (|*r*| > 0.8, and *p* < 0.01) among bacterial and microeukaryotic ASVs in the ET and ST regions, respectively. The observed modularity, average clustering coefficient, and average path length in the networks were higher than those in the corresponding Erdös–Réyni random networks, suggesting that all networks exhibited “small-world” properties and non-random modular structures (Supplementary Table S3). All networks were divided into 4 major modules that accounted for 80 and 91.14% of the bacterial community (in ET and ST, respectively) and 88.54 and 88.52% of the microeukaryotic communities (in ET and ST, respectively). Hydrological factors like Outflow and Inflow, as well as water temperature (WT), displayed high degrees within these networks, indicating that hydrological factors and WT had strong relationships with microbial ASVs compared to other environmental parameters (Figure 4A). Bacterial and microeukaryotic networks in the ET region contained fewer nodes and edges compared to the ST region (Figure 4A) and demonstrated significantly lower values of degree betweenness centrality and closeness centrality (Figure 4B). This suggested a more simplified structure of microbial associations in the ET than in the ST region.

We compared network stability between both regions and found that the natural connectivity of bacterial and microeukaryotic networks was lower in the ET than in the ST region, indicating reduced network robustness for both bacteria and microeukaryotes in the ET region (Figure 4C). Cohesion analysis further assessed taxa associations resulting from positive and negative ASV interactions. In both bacterial and microeukaryotic communities, regardless of positive or negative relationships, association strength was significantly lower in the ET than in the ST region (Figure 4D). This indicated that cooperative (positive cohesion) and competitive interactions (negative cohesion) were poorly developed among microbes in the ET region, resulting in lower network stability in the ET than in the ST region.

In addition, we found that ASVs with a higher degree of centrality exhibited stronger correlations with hydrological factors and WT. There was a higher slope in those linear relationships in the ET than in the ST region; however, in the ST region, Outflow parameters did not meet the network correlation threshold criteria (Figure 4E). This indicates that hydrological factors and WT affect the coexistence networks of bacteria and microeukaryotes, with a greater impact in the ET than in the ST region.

In the bacterial community networks, Xanthomonadaceae, Moraxellaceae and Micrococcaceae exhibited high degree of centrality in the ET modules, while Ilumatobacteraceae, Sphingomonadaceae, and Mycobacteriaceae showed high degree of centrality in the ST modules. In microeukaryotic networks, Metazoa and Gyrista had high degree of centrality in the ET modules, while Chlorophyta showed high degree of centrality in most ST modules (Supplementary Tables S4–S7). This indicated that Xanthomonadaceae, Moraxellaceae, Micrococcaceae, Metazoa, and Gyrista played key roles in maintaining coexistence in the high-velocity ET region. In contrast, Ilumatobacteraceae, Sphingomonadaceae, Mycobacteriaceae, and Chlorophyta were more important for maintaining coexistence in the low-velocity ST region, as nodes with a higher degree of network centrality were crucial for maintaining taxa coexistence.

### 3.5 Associations among environment, community diversity, and ecological niche

**Figure 5:**
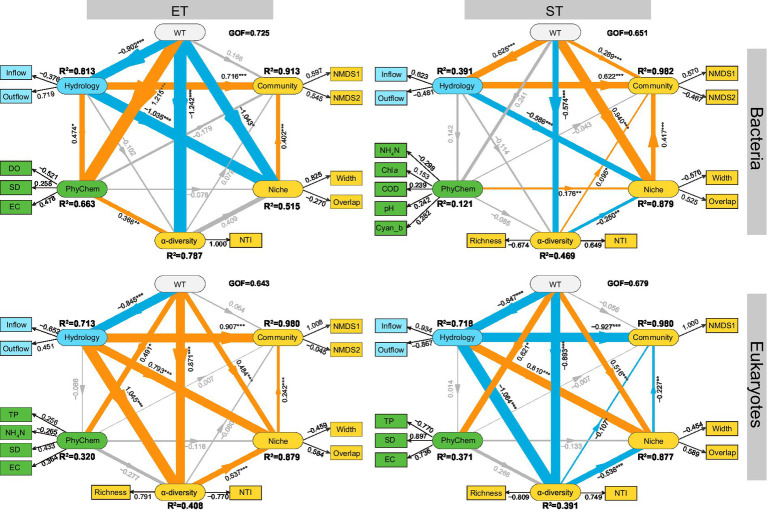
Predicted partial least squares path modeling (PLS-PM) delineated both direct and indirect relationships between environmental drivers and the diversity and niche indices of bacterial and microeukaryotic communities in the ET and ST regions. The model is visualized with rounded rectangles representing the structural model, while corresponding measurement models are indicated by boxes. In the structural model, paths are shown with lines, with adjacent values representing the magnitude of path coefficients derived from PLS regression. *R*^2^ values for all endogenous latent variables are included within the rounded rectangles. In the measurement model, the values signify the weights, linking a latent variable to its indicators. The diagram displays the refined model’s post-diagnostic procedures. Positive and negative effects are depicted by orange and blue lines, respectively, with gray lines indicating non-significant paths (*p* > 0.05). The thickness of the line correlated with the absolute values of the path coefficients. The pseudo goodness-of-fit (GOF) values surpassing 0.64 confirms the models’ robust fit. The model includes variables such as Water Temperature (WT), Physicochemical Factors (PhyChem), Monthly Net Inflow Volume (Inflow) and Outflow Volume of Lake Taihu (Outflow), Dissolved Oxygen (DO), Secchi Depth (SD), Electrical Conductivity (EC), Total Phosphorus (TP), Ammonia Nitrogen (NH_4_N), Chlorophyll *a* (Chl*a*), Permanganate Index (COD), Cyanobacterial Biomass (Cyan_b), Nearest Taxon Index (NTI), Niche Width (Width), and Niche Overlap (Overlap), alongside NMDS1 and NMDS2 from non-metric multidimensional scaling of microbial communities.

The PLS-PM model revealed that WT had a minimal direct impact on community composition, but exerted a stronger and more significant influence on *α*-diversity and ecological niches in both regions (Figure 5). Hydrological factors (Inflow and Outflow) significantly affected community composition and niches in both regions and strongly influenced the α-diversity of microeukaryotic communities, with path coefficients of 1.045 in ET and − 1.064 in ST. For bacterial communities, hydrological factors had a greater impact on community composition and niches in the ET (path coefficients were 0.716 and − 1.035, respectively) than in the ST region (path coefficients were 0.622 and − 0.586, respectively). Conversely, in microeukaryotic communities, hydrological factors had a slightly greater impact in the ST region. Across both regions and kingdoms, the influence of physicochemical factors on α-diversity, *β*-diversity, and niches was minimal (Figure 5). These results indicated that seasonality (WT) and water flow significantly influenced the diversity and ecological niches of bacterial and microeukaryotic communities in the Lake Taihu Basin.

## 4 Discussion

Bacteria and microeukaryotes are critical for ecological processes in river ecosystems (Siriarchawatana et al., 2024). Our current knowledge of how bacteria and microeukaryotes responded to different intensities of water flow disturbances along the urban river–lake continuum was quite limited. In our study, we provided molecular insights into the comprehensive characteristics of bacterial and microeukaryotic communities under differing water flow and temperature disturbance in two interconnected urban river–lake systems of the Taihu basin. Our results revealed that biodiversity, community construction, niche breadth, and co-occurrence patterns of bacteria and microeukaryotes were significantly influenced by the intensity of water flow disturbances. This notion highlighted the significance of understanding the mechanisms of bacterial and microeukaryotic succession in changing environments for elucidating ecosystem recovery processes and watershed management following environmental disturbances.

### 4.1 Effects of hydrological factors on microbial community richness and structure

Our findings indicated that hydrological conditions, specifically the inflow and outflow water flow of Lake Taihu, were the primary factors influencing community diversity and composition of both bacteria and microeukaryotes. Moreover, significantly higher richness and NTI diversity indices were observed in the higher water flow ET region than in the ST region with reduced water flow. This indicated that the microbial community in the ET region comprised closer phylogenetic relationships. This might be because the microbial community in the ET region had undergone a stronger environmental filtering (Fillinger et al., 2021), enabling certain microbial species with specific traits to survive and proliferate more easily in this habitat (Gweon et al., 2021). This environmental filtering could be due to the higher disturbances in the riverine environment of the ET region, requiring microbes to possess specific adaptations, leading to evolutionary convergence.

Water flow can alter the effects of intensity of habitat disturbance intensity on microbial dynamics (Wheeler et al., 2019). In the ET region, larger and irreversible discharge rates likely increased the degree of disturbances, enhancing environmental heterogeneity. Conversely, in the ST region, gentler and more reversible water flow resulted in a lower degree of disturbances, causing a relatively stable microbial growth environment with greater similarity among environmental factors (Figure 3D). These differences in disturbance May significantly impact the structure of microbial communities within aquatic ecosystems (Yang et al., 2017), resulting in a higher dissimilarity of bacterial and microeukaryotic communities in the ET than ST region (Figure 2D). This was in line with previous findings, highlighting that hydrodynamic changes, e.g., flow velocity, had profound impacts on algal dynamics in riverine and reservoir ecosystems (Chen et al., 2023; Li et al., 2024; Tan et al., 2022; Yang et al., 2022).

Although both bacterial and microeukaryotic communities were significantly impacted by the examined hydrological variables, according to our NMDS analysis, their overall dynamics was better explained by seasonality (Supplementary Figure S2). Hierarchical partitioning and random forest analyses revealed a strong co-influence of temporal and environmental factors, such as WT, DO, EC, and pH, which can be at least partially attributed to the seasonal variability of the examined variables (Supplementary Figure S4). This suggested season-driven habitat filtering effects on the microbiota. Our findings implied a strong interplay between temporal, spatial, and environmental factors in shaping microbial communities in river–lake systems, similar to patterns observed in natural lakes (Ann et al., 2022; Liu et al., 2024; Zhang et al., 2023b; Zhu et al., 2019).

### 4.2 Effects of hydrological factors on microbial community assembly

Both the neutral community model (NCM) and Null model suggested that the assembly of bacterial and microeukaryotic communities in both ET and ST regions was predominantly influenced by stochastic processes. Furthermore, stochastic processes exerted a stronger impact in the high-flow ET region compared to the low-flow ST region, whereby dispersal limitation and ecological drift were identified as significant processes (Figure 3). Flow direction and intensity can affect microbial migration and dispersal and thus shape microbial diversity, leading to profound differences in community structure between ET and ST regions (Carrara et al., 2012; Tonkin et al., 2018). In the ST region, high rainfall in the southwestern cities during rainy seasons can lead to pronounced differences in water levels between the rivers and Lake Taihu, often reversing the flow from rivers into the lake. This hydrological condition of outflow and backflow potentially enhanced the passive dispersal of microbial communities. Compared to higher flow rates and a stable outflow direction in the ET region, the relatively moderate water flows in the ST region May reduce environmental disturbances, leading to increased species sorting and reduced contributions from stochastic processes (Figures 3C,D). This finding aligned with previous mesocosm experiments (Bier et al., 2022). Additionally, flow alterations May alter nutrient conditions, water temperatures and quantity of downstream areas, shaping the structure of downstream microbial communities (Lambert et al., 2016; Li C. et al., 2023b; Lynch et al., 2019; Maavara et al., 2020).

Headwaters serve as important sources of biodiversity, and combined with directional flow processes, May constantly influence the structure of downstream microbial communities (Geng et al., 2024; Meyer et al., 2007). The Local Contribution to Beta Diversity (LCBD) results indeed indicated that in the ET region, where only outflow takes place, Lake Taihu’s headwater significantly contributed to the downstream community structure. Conversely, this phenomenon was not observed in the ST region, where water can flow both out and into the lake (Supplementary Figure S6). This suggested that headwaters with distinct terrestrial habitats might lead to variations in the community structure of riverine microbial communities at the local scale, consistent with findings from studies on bacterial communities in the high-altitude Ili River (Geng et al., 2024) and urban Tsurumi River (Yokoyama and Kikuchi, 2023). Today, it is widely accepted that microbial community similarity decreases as the distance between sampling sites increases, and vice versa (Hayden and Beman, 2016; Martiny et al., 2006). Thereby, in rivers, geographic distance was particularly linked to the mass effect, bacterial growth competition, and predation (Read et al., 2015).

### 4.3 Effects of hydrological factors on microbial co-occurrence networks

Variations in the intensity of flow disturbance May result in different shear flow, which in turn alter the contact or adhesion among microbes, subsequently reducing their co-occurrence associations and leading to distinct composition and dynamics of microbial communities (Geng et al., 2024; Wheeler et al., 2019). Shear flow occurs when fluid volumes with different velocities, e.g., a layer of warm surface water over colder deep water, flow past each other. Such surface shear flow impacted microbial organisms near or on those surfaces (Geng et al., 2024; Wheeler et al., 2019). Moreover, flow velocity has been reported to destabilize the co-occurrence network stability of bacterial and microeukaryotic communities (Mu et al., 2021). These findings are consistent with our results. For instance, in the ST region water flow disturbances were relatively mild, whereas intense water flow disturbances in the ET region reduced the complexity and stability of the coexistence networks of both bacteria and eukaryotic microorganisms (Figure 4). Future work should include larger-scale, multi-habitat studies for further validation to enhance the biological relevance of monitoring and assessing interconnected river–lake ecosystems.

### 4.4 Differences in tolerance to intensity of flow disturbances between bacterial vs. eukaryotic microorganisms

Unlike bacteria, microeukaryotic communities in river–lake systems remain largely unexplored. Stochastic processes, primarily dispersal limitation and drift, played a dominant role in the assembly of both bacterial and microeukaryotic communities. However, these processes had a greater impact on microeukaryotic communities than on bacteria. Microeukaryotes exhibited lower migration and dispersal rates, making their community assembly more susceptible to dispersal limitations than bacteria (Figures 3A–C and Supplementary Figure S4). One reason could be the generally larger size of microeukaryotes compared to bacteria, which increased their resistance to water flow and settling rates (Blais et al., 2024). Additionally, due to their more complex cellular structures, microeukaryotes had slower reproduction rates than bacteria, resulting in lower diffusion and migration efficiencies (Yang et al., 2023). Also, the coexistence networks of microeukaryotes were more stable and complex than bacteria, as evidenced by increased node and edge numbers (Figure 4). A possible reason was that microeukaryotic networks May involve more complex interactions, such as symbioses, predation, and mutualism, contributing to such network complexity and stability (Faust and Raes, 2012; Mathis and Bronstein, 2020). Previously, it has been shown that these interactions were crucial for maintaining community structure and function, especially under water flow disturbances or environmental stress (Palmer and Ruhi, 2019). Compared to bacterial communities, water flow significantly affected the alpha and beta diversity of microeukaryotic communities. Furthermore, water flow significantly reduced the ecological niche width of bacteria, but enhanced those of microeukaryotes (Figure 5). Whereas bacteria often adapted to environmental disturbances through rapid reproduction and metabolic flexibility (Wood et al., 2023), microeukaryotes can use structural and behavioral adaptations (e.g., using cilia or flagella for movement) to manage challenges posed by changes in water flow. Disturbance conditions created by strong water flow, thus, can reduce competition and predator pressure, allowing microeukaryotes to successfully colonize newly available niches. Previously, microcosm experiments had demonstrated that with increasing intensity of environmental disturbance, microeukaryotes experienced an accelerated tempo of competitive exclusion (Violle et al., 2010) and thus were more sensitive to environmental changes than bacteria (Li C. et al., 2023b). Such dynamics could lead to quicker shifts in community composition and diversity.

## 5 Conclusion

In this study, we analyzed the dynamics of bacterial and microeukaryotic microbial communities in the interconnected river–lake systems of East (ET) and South Lake Taihu (ST) under different intensities of flow disturbances using amplicon sequencing and multiple statistical analyses. Diversity, community composition and assembly, as well as co-occurrence relationships of both bacteria and microeukaryotes in the ET region with higher flow disturbance, were significantly distinct from those in the ST region. Stochastic processes, mainly dispersal limitation and drift, dominated community assembly, with the proportion of dispersal limitation increasing alongside flow intensity. Increasing water flow disturbances reduced the ecological niche width and stability of the coexistence networks of both bacteria and microeukaryotes. Compared to bacteria, microeukaryotes were more sensitive to changes in water flow intensity. These results were particularly relevant in the context of Lake Taihu, which had been plagued by frequent cyanobacterial blooms in recent years. These blooms, driven by nutrient over-enrichment and hydrological changes, had led to severe ecological and public health challenges. Understanding the microbial dynamics under varying flow conditions is crucial for managing these blooms, as the interplay between microbial community structure and hydrological factors directly influences the occurrence and persistence of cyanobacterial blooms. Our findings expand our understanding of the diversity, construction, and ecological mechanisms of bacterial and microeukaryotic communities under differing flow conditions along the river–lake continuum, offering critical insights into how these processes can be leveraged to mitigate the impacts of cyanobacterial blooms. These findings have far-reaching implications for mitigation and management strategies of human alterations of flow conditions, especially in connected river–lake ecosystems like Lake Taihu, where the control of cyanobacterial blooms is a key environmental and public health priority.

## Data Availability

The sequencing data was deposited in the Sequence Read Archive database of NCBI with a BioProject accession PRJNA1138527.

## Footnotes

^1^ https://www.tba.gov.cn/

^2^ http://www.r-project.org

^3^ https://github.com/Hy4m/linkET
