# Supplementary material for: Differential microbiome features in lake–river systems of Taihu basin in response to water flow disturbance
Source: Front Microbiol. 2024 Sep 30;15:1479158. doi: 10.3389/fmicb.2024.1479158 (PMC11475019; doi:10.3389/fmicb.2024.1479158)
Supplement: Supplementary file 2 [file Image_1.pdf]

Supplementary Materials for

Differential Microbiome Features in Lake-River Systems of Taihu Basin in Response to Water Flow Disturbance

Peng Xiao *et al.*

*Corresponding author. Email: renhui.li@wzu.edu.cn, yailing99@163.com

This PDF file includes:

Figure S1 to S6

Other Supplementary Materials for this manuscript include the following:

Tables S1 to S7

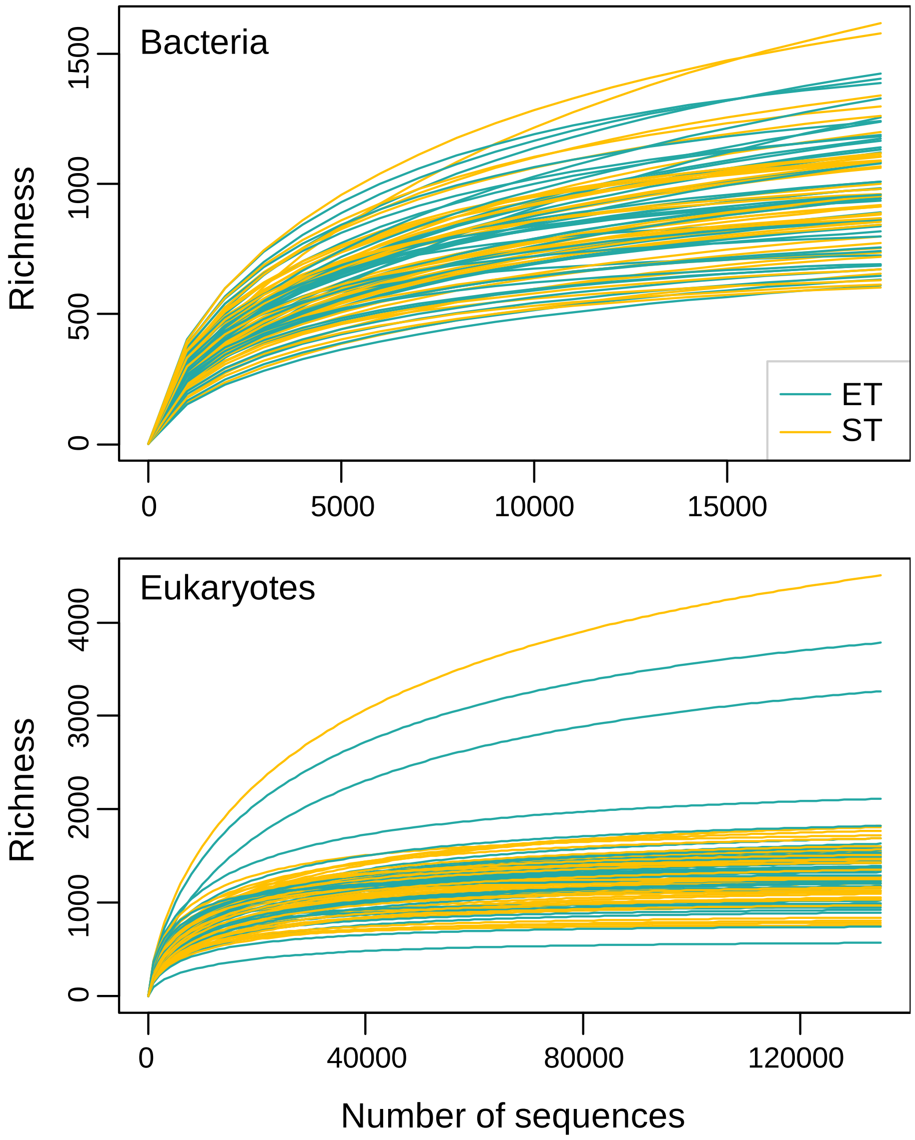

Fig. S1. The rarefaction curve of bacterial and microeukaryotic richness.

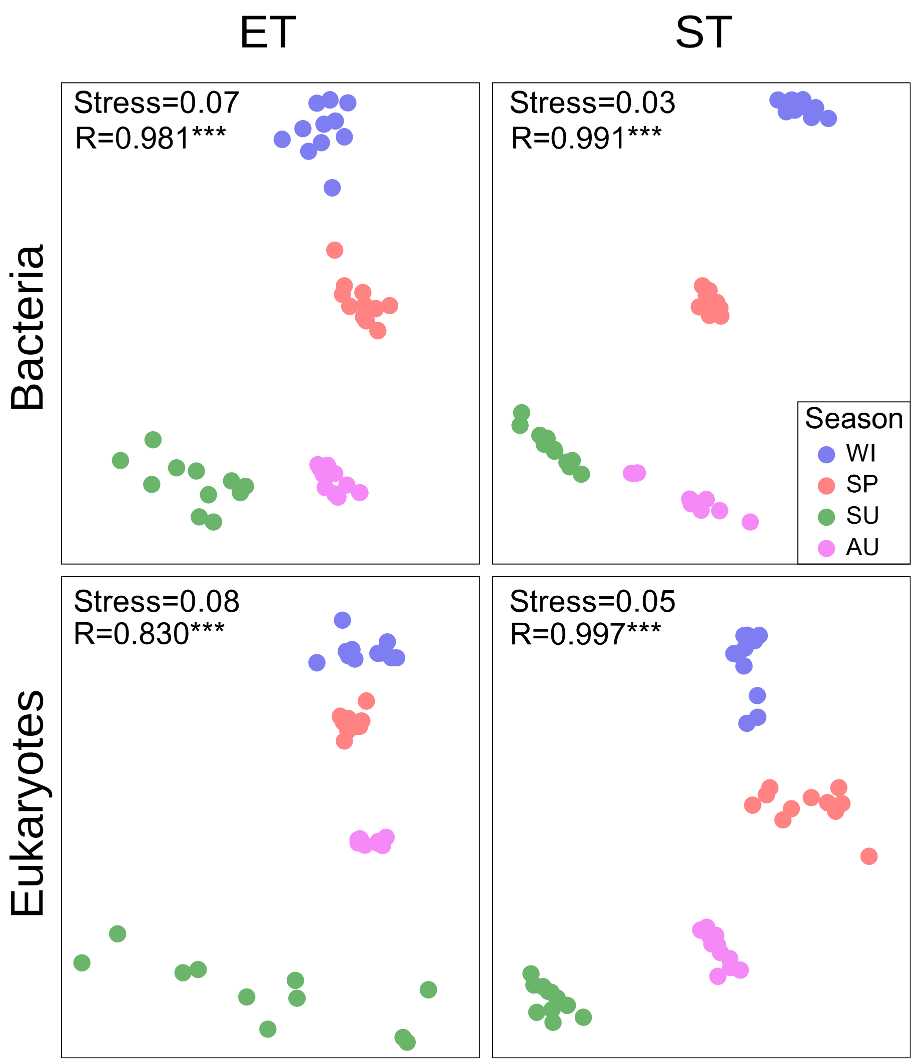

Fig. S2. Seasonal variation of bacterial and microeukaryotic communities in the ET and ST regions. R represented the ANOSIM R values, ****P*=0.001. WI, winter; SP, spring; SU, summer; AU, autumn.

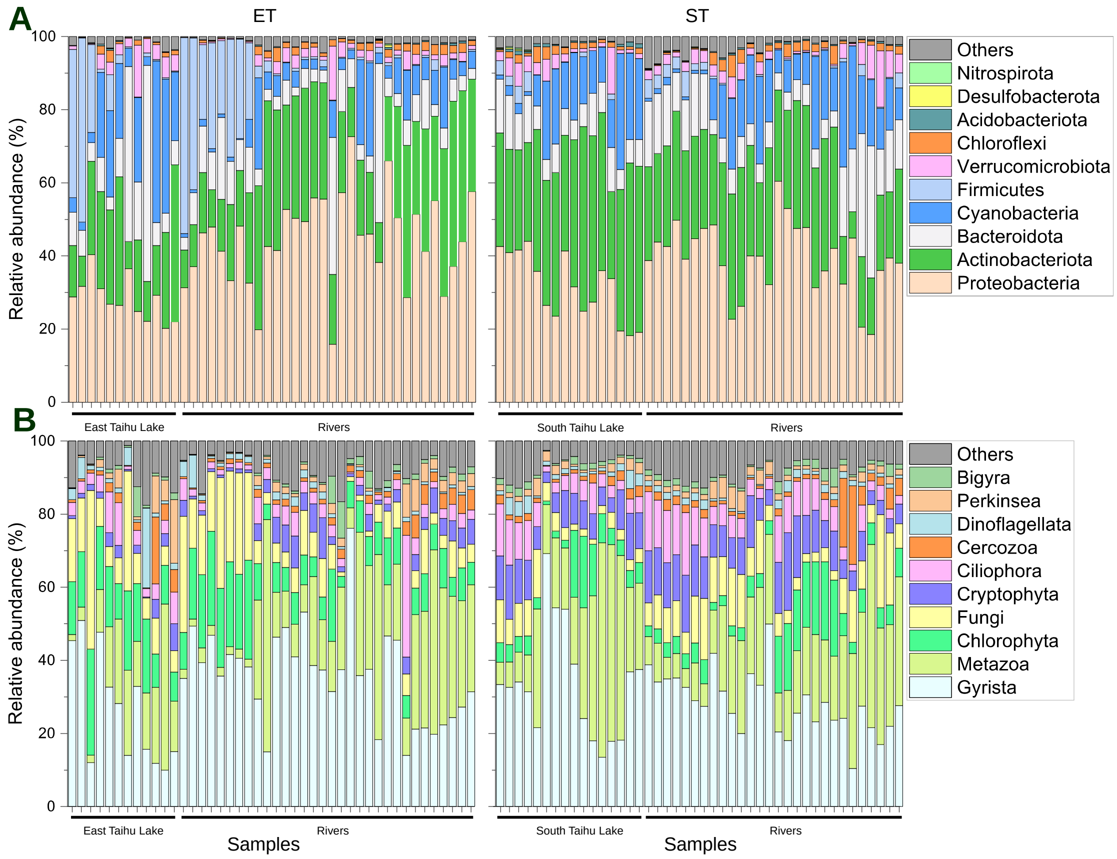

Fig. S3. Bacterial (A) and microeukaryotic (B) community composition at the phylum level.

Only the 11 dominant phyla of bacteria and microeukaryotes were shown, respectively.

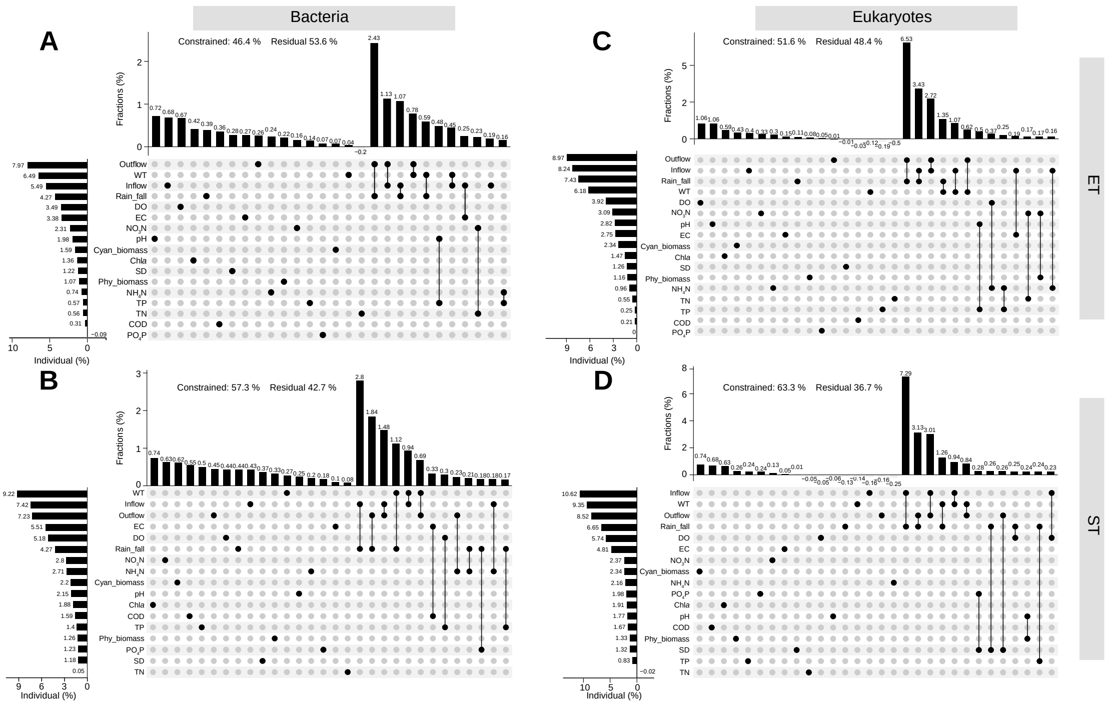

Fig. S4. Upset matrix layout of hierarchical partitioning results to show the relative importance of environmental factors on bacterial and microeukaryotic microbial community in the ET and ST regions. In the dot-matrix plot on the right, rows represent individual environmental factors. Each column's isolated black dot depicts the marginal effect of a specific factor, while lines connecting dots indicate shared effects among factors. The top column diagram displays the percentage of variation each component explained, derived from variation partitioning. The left column diagram illustrates the individual effect of each factor, calculated from hierarchical partitioning, as the sum of its marginal effect and average shared effect with other factors.

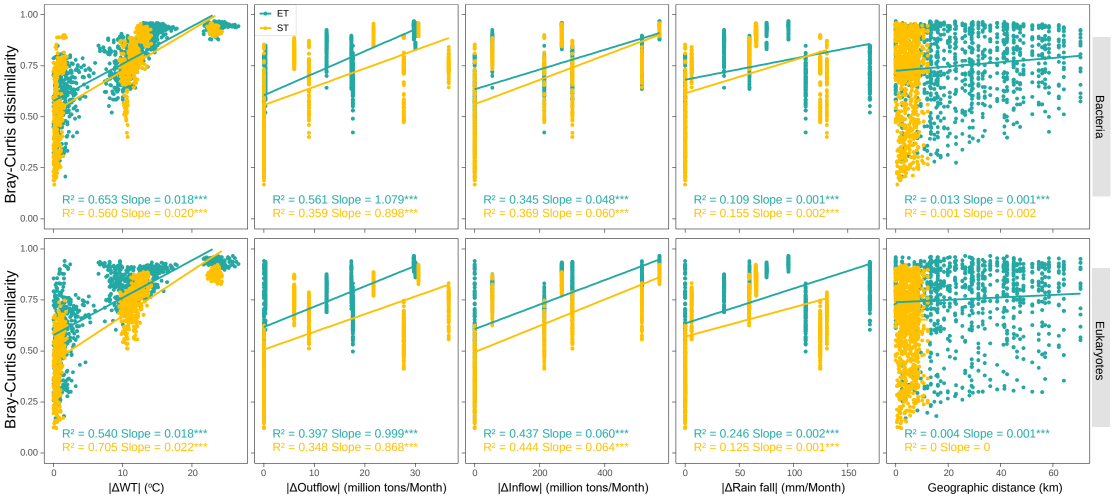

Fig. S5. The influence of environmental factors on Bray-Curtis dissimilarity of bacterial and microeukaryotic microbial communities in ET and ST regions (****P* < 0.001).**

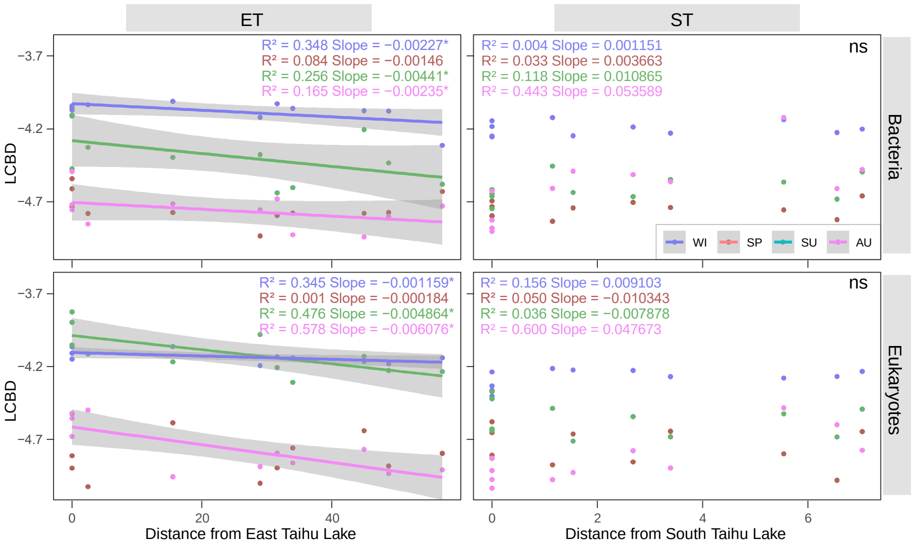

Fig. S6. Local contribution to beta diversity (LCBD) of different sampling sites of bacterial and microeukaryotic communities in the ET and ST regions. *, *P* < 0.10; ns, non-significant.
